# Effects of Multi-Shell Free Water Correction on Glioma Characterization

**DOI:** 10.3390/diagnostics11122385

**Published:** 2021-12-17

**Authors:** Lea Starck, Fulvio Zaccagna, Ofer Pasternak, Ferdia A. Gallagher, Renate Grüner, Frank Riemer

**Affiliations:** 1 Department of Physics and Technology, University of Bergen, N-5007 Bergen, Norway; renate.gruner@uib.no; 2 Mohn Medical Imaging and Visualization Centre (MMIV), Department of Radiology, Haukeland University Hospital, University of Bergen, N-5021 Bergen, Norway; frank.riemer@helse-bergen.no; 3 Department of Biomedical and Neuromotor Sciences, University of Bologna, 40125 Bologna, Italy; f.zaccagna@gmail.com; 4 IRCCS Istituto delle Scienze Neurologiche di Bologna, Functional and Molecular Neuroimaging Unit, Bellaria Hospital, 40139 Bologna, Italy; 5 Department of Psychiatry, Brigham and Women’s Hospital, Harvard Medical School, Boston, MA 02215, USA; ofer@bwh.harvard.edu; 6 Department of Radiology, Brigham and Women’s Hospital, Harvard Medical School, Boston, MA 02215, USA; 7 Department of Radiology, University of Cambridge, Cambridge CB2 0QQ, UK; fag1000@cam.ac.uk; 8 Cancer Research UK Cambridge Institute, University of Cambridge, Cambridge CB2 0RE, UK

**Keywords:** glioma, free water, tumor characterization

## Abstract

Diffusion MRI is a useful tool to investigate the microstructure of brain tumors. However, the presence of fast diffusing isotropic signals originating from non-restricted edematous fluids, within and surrounding tumors, may obscure estimation of the underlying tissue characteristics, complicating the radiological interpretation and quantitative evaluation of diffusion MRI. A multi-shell regularized free water (FW) elimination model was therefore applied to separate free water from tissue-related diffusion components from the diffusion MRI of 26 treatment-naïve glioma patients. We then investigated the diagnostic value of the derived measures of FW maps as well as FW-corrected tensor-derived maps of fractional anisotropy (*FA*). Presumed necrotic tumor regions display greater mean and variance of FW content than other parts of the tumor. On average, the area under the receiver operating characteristic (ROC) for the classification of necrotic and enhancing tumor volumes increased by 5% in corrected data compared to non-corrected data. FW elimination shifts the *FA* distribution in non-enhancing tumor parts toward higher values and significantly increases its entropy (*p* ≤ 0.003), whereas skewness is decreased (*p* ≤ 0.004). Kurtosis is significantly decreased (*p* < 0.001) in high-grade tumors. In conclusion, eliminating FW contributions improved quantitative estimations of *FA*, which helps to disentangle the cancer heterogeneity.

## 1. Introduction

Glial tumors are the most frequent malignant primary brain tumors, with incidence rates of 5.4/100,000 in Europe and 7.1/100,000 in the United States [1,2,3]. Gliomas are highly heterogeneous and infiltrative, limiting a complete surgical removal and successful treatment. Overall, the proportion of patients that fully recover is approximately 7.9% [3]. Gliomas can occur at any age, but when comparing to individuals under 20 years, gliomas are approximately 1.5 times more common in those aged over 20 years [4] and more than 10 times more common in those aged over 60 years [3]. Magnetic resonance imaging (MRI) retains a key role for diagnosis, treatment planning, and response monitoring of gliomas using both conventional and more advanced imaging techniques [5,6,7]. Based on morphological proton imaging, the tumor is commonly subdivided into different sub-regions: solid tumor, enhancing, non-enhancing, or peritumoral areas [8,9,10,11].

Diffusion tensor imaging (DTI) is an advanced technique that can estimate fractional anisotropy (*FA*) and mean diffusivity (*MD*), describing properties of the underlying tissue microstructure. There is still no consensus on the optimal methodology for processing DTI data; consequently, their ability to characterize gliomas, particularly regarding tumor grade, is still a matter of debate [8,9,10,12]. The most reported metrics are mean estimates of *MD* and *FA*, although statistical measures other than the mean could be of interest [10,11]. Based on eigenvalues and eigenvectors of the estimated diffusion tensor in each imaged voxel, tractography is often performed to create a three-dimensional visualization of fiber tracts [13,14].

High-grade gliomas typically spread outside the boundaries of the contrast enhancing lesion, within the surrounding peritumoral non-enhancing region of signal change. This spread may cause a reduction of *FA*, due to disrupted fiber tracts [15,16], but this is difficult to differentiate from the similar reduction of *FA* caused by vasogenic edema also abundant in the peritumoral region [17]. Vasogenic edema is mostly composed of free water (FW), and the fast diffusion of water molecules within edematous tissues will cause a more rapid diffusion signal decay than typically observed in tissues where water is more hindered or restricted. As a result, the DTI measures in edematous regions are biased by the FW contribution and reflect a mixture of edema and tissue.

One means of extracting the isotropic diffusion signal contribution from the edema is through advanced mathematical modelling [18,19,20,21]. A two-compartmental model accounting for free water contribution was first proposed by Pierpaoli and Jones [22]. The model assumes that the measured diffusion attenuation signal is the sum of attenuation in the two compartments and that these compartments have different diffusion tensors. In this study, an FW modelling approach proposed by Pasternak et al. [18] is applied in gliomas to improve tumor characterization. Since tumor heterogeneity is a hallmark of different biological and metabolic microenvironments, an in vivo assessment of heterogeneity may help, for instance, in determining treatment response, distinguishing true disease progression from pseudo-progression [23,24,25]. Necrosis is the result of a reduction in cell density [26,27], and increased FW content could provide a basis for a more detailed characterization of the expansion of necrosis. Improving the characterization of gliomas may also improve non-invasive tumor grading, as well as allow more accurate treatment planning. For example, several studies have reported increased fiber tracking capabilities after FW correction [28,29,30,31,32], indicating that FW correction may improve treatment planning.

To understand the impact of the advanced modelling in gliomas, diffusion tensor data are analyzed in the current study both with and without the free water elimination. Receiver operator characteristic (ROC) curves are estimated to investigate the classification of tumor sub-regions in high-grade tumors. Furthermore, potential differences between corrected and non-corrected *FA* estimations are evaluated in low- and high-grade tumors.

## 2. Materials and Methods

### 2.1. Data Collection

Twenty-six treatment-naïve glioma patients (11 males, 15 females; 52 ± 18 years, range 21–78 years), part of a cohort previously recruited for prospective, ethically approved studies, were included in this retrospective study. Patient characteristics are summarized in Table 1. MRI was carried out as part of routine surgical planning at Addenbrooke’s Hospital, Cambridge University NHS Foundation Trust. Subjects included in this study matched the following criteria: glioma confirmed on histology, presence of structural MR imaging, multiple b-value diffusion imaging, and binarized masks of tumor volume. Written and informed consent was obtained from all participants and the study was approved by the NRES Committee East of England, Cambridge 2 ethics committee.

Data were collected on a 3 T MRI system (Discovery MR750, GE Healthcare, Waukesha, WI, USA) using a 12-channel head coil. Structural T_1_ -weighted (T_1_w) images (256 × 256 matrix size, slice thickness 1.5 mm, FOV 240 mm × 240 mm) were acquired using an inversion-prepared fast 3D spoiled gradient echo sequence (*TR/TE/TI/FA* = 8.2 ms/3.2 ms/450 ms/12°). T_2_ -weighted (T_2_w) images (320 × 320 matrix size, slice thickness 1.2 mm, FOV 240 mm × 240 mm) were acquired using a 3D spin echo sequence (*TR/TE* = 2500 ms/79 ms).

Multi-shell DWI were acquired as follows with a pulsed-gradient spin-echo echo-planar imaging (EPI) sequence: b-values 0, 90, 150, 500, and 1000 s/mm^2^; 256 × 256 matrix size; slice thickness 2 mm; FOV 220 mm × 220 mm; *TR/TE* = 2000 ms/80 ms. Diffusion acquisitions were performed in 8 directions in each shell. Low b-value shells were selected because, with a diffusivity of 3.00 × 10^−3^ mm^2^/s, FW is fast-diffusing compared to normal brain tissue, which has a diffusivity of approximately 0.8 × 10^−3^ mm^2^/s. Hence, lower b-values are better suited to estimate FW than higher b-values.

**Figure 1:**
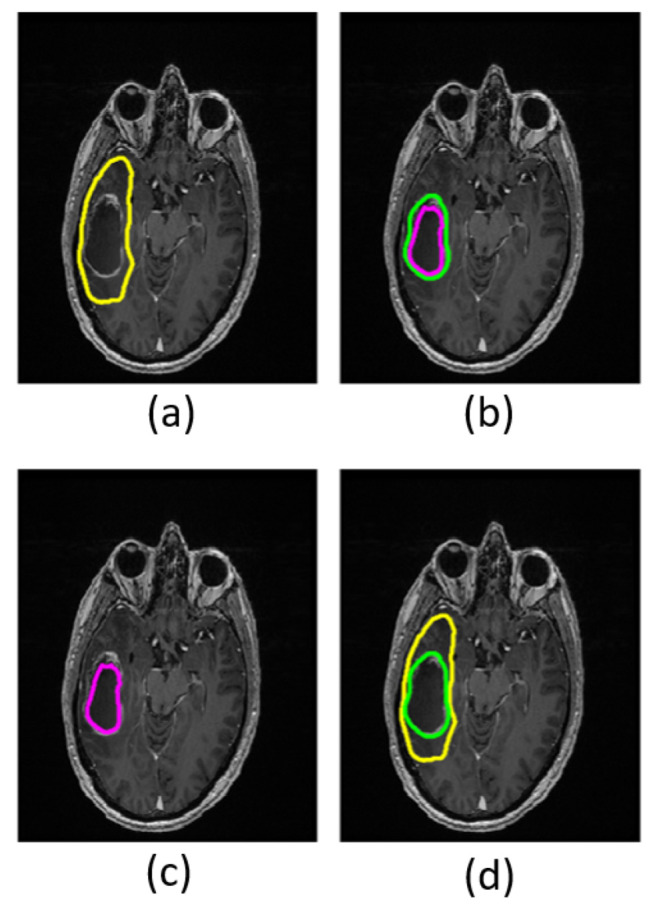
Masks in a grade IV patient overlaid on structural T_1_w images. The yellow boundary shows the total tumor region (**a**); the area between the green and purple boundaries shows the enhancing tumor region (**b**); the area within the purple boundary shows necrotic tumor region (**c**); and the area between the yellow and green boundaries shows the non-enhancing tumor region (**d)**.

Based on the T_1_w- and T_2_w-images, a neuroradiologist with 8 years of experience manually outlined regions of interest (ROIs) encompassing the entire lesion, including the peritumoral region (total tumor volume), the enhancing lesion (enhancing tumor region), and the non-enhancing, presumed necrotic core (necrotic tumor region) (Figure 1). ROIs for the non-enhancing tissue (non-enhancing tumor region) were calculated by subtracting the enhancing tumor region and necrotic tumor region from the total tumor region. The tumor grades were confirmed by histology according to the 2016 WHO criteria [33,34].

### 2.2. Data Modelling

Each voxel is modelled as a weighted sum of two compartments, one compartment composed of free water (FW compartment), the other containing water in the vicinity of tissue structures that hinder or restrict water diffusion (tissue compartment). The diffusivity of the FW compartment is isotropic and fixed to 3.00 × 10^−3^ mm^2^/s, which is the diffusivity of FW at body temperature [35]. The data are modelled using a regularized fit [18], implementing a Euclidean tensor metric [36], and initialized with tensors with MD of 0.6 × 10^−3^mm^2^/s. The fractional volume of the FW compartment, *f*, and the diffusion tensor modelling the tissue compartment are estimated. *FW* maps provide the voxel-wise *f* value, and the tissue compartment tensor is decomposed to eigenvalues to calculate free water corrected *FA*.

### 2.3. Data Processing and Parameter Extraction

Motion correction was performed using the FSL toolbox (version 6.0.1, University of Oxford, Oxford, UK) [37]. Skull stripping was performed prior to fitting of parameters [38].

Both regular tensor fits and free water corrected multi-shell tensor fits were computed from the data. Regular tensor fitting was applied to a single shell with b-value 1000 s/mm^2^ using FSL [37]. Free-water-corrected tensor fitting was applied to the multi-shell diffusion data (b-values 90, 150, 500, and 1000 s/mm^2^). B0 images were registered to the T_1_w images using a normalized mutual information cost function and a 4th degree b-spline interpolation in SPM12 (version 7771, University College London, London, UK) [39]. The transformation was then applied to all calculated maps, including the *FW* maps, FW-corrected *FA* (*FAt)*, and non-corrected *FA* (*FA*) maps. Measurements were extracted from the total tumor region and non-enhancing regions using (1) *FA* maps derived with regular tensor fitting, (2) *FAt* maps derived with free water corrected tensor fitting, and (3) *FW* maps derived with free-water-corrected tensor fitting. Map-derived ROI summary variables including mean and variance, 25th and 75th quantile, median, skewness, kurtosis, and entropy of the *FA* and *FW* map were computed.

FW correction failed in one out of the 15 grade IV patients, due to corrupted DICOM images in the low b-value diffusion images.

### 2.4. Statistical Analysis

To test whether tumor volumes, i.e., necrotic and enhancing volumes, and enhancing and non-enhancing volumes can be separated based on parameter means within these volumes after FW correction, ANOVA testing with Tukey’s post hoc test was performed. To test whether conventional mean *MD* that is not corrected for FW can differentiate between tumor regions, the same ANOVA test with Tukey’s post hoc test was performed using non-corrected data. Receiver operator characteristic (ROC) curves were computed by fitting a logistic regression model to labelled image voxels to evaluate the ability of FW-corrected data to classify voxels to their assigned tumor volume.

The effect of FW correction on the various tumor volumes (enhancing, necrotic, non-enhancing, and total tumor volumes) were investigated using a paired *t*-test.

To assess group differences, the patient cohort was separated into low-grade (grades I and II) and high-grade (grades III and IV) tumors, and differences were investigated using paired *t*-tests.

Results were deemed statistically significant for *p* < 0.05, and *p*-values were computed with *t*-tests adjusted for multiple comparisons using the Bonferroni method.

## 3. Results

### 3.1. Characterizing Tumor Sub-Regions

**Figure 2:**
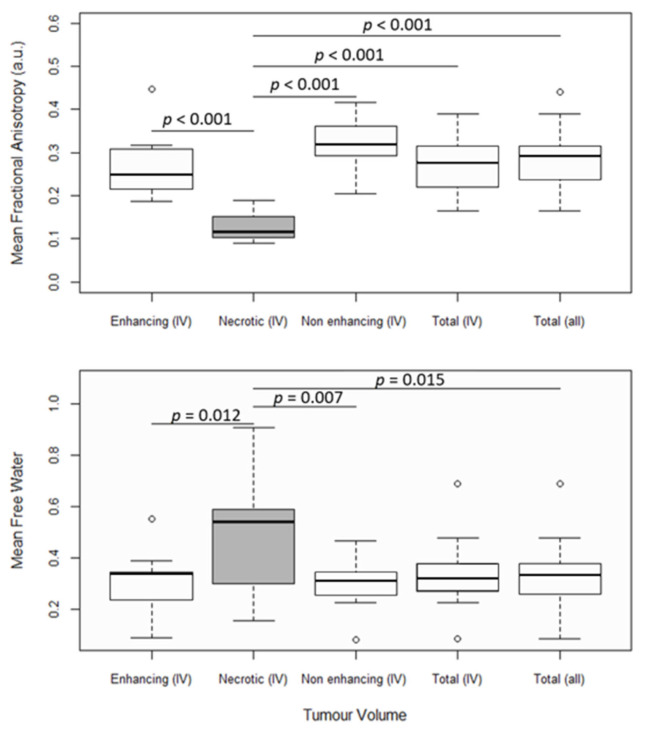
FW-corrected fractional anisotropy (*FAt*) and mean free water (*FW*). The first four box and whisker plots from the left show the mean parameter values in enhancing tumor regions, necrotic tumor regions, non-enhancing tumor regions, and total tumor regions from grade IV patients. The fifth box and whisker plot shows the mean parameter values in total tumor regions from all patients. Significantly different distributions are marked with their corresponding *p*-values. Boxes with white backgrounds do not differ significantly from other boxes with white backgrounds. Statistics were performed with ANOVA tests and Tukey’s post hoc test at a significance threshold of *p* < 0.05.

There is a larger variance in the distribution of the mean *FW* in the necrotic tumor region than in other parts of the tumor (Figure 2), and the mean *FW* is significantly higher in the necrotic region (*FW_mean_* = 0.50) than in both the enhancing (*FW_mean_* = 0.30) and non-enhancing (*FW_mean_* = 0.30) regions in grade IV patients (*p* ≤ 0.015), as well as in the total tumor volume region across all patients (*FW_mean_* = 0.33). Mean *FAt* is significantly lower (*p* ≤ 0.001) in the necrotic tumor region (*FAt_mean_* = 0.13) than in the other defined tumor regions (*FW_mean_* ≤ 0.27). Measurements of *FA* and *FW* from the enhancing, non-enhancing, and total tumor regions do not differ statistically significantly from one another. Although there may be tendencies toward higher *MD* in the necrotic region compared to non-necrotic regions, conventional *MD* that is not corrected for FW does not significantly differentiate between tumor regions (Supplementary Material, Figure S1).

**Figure 3:**
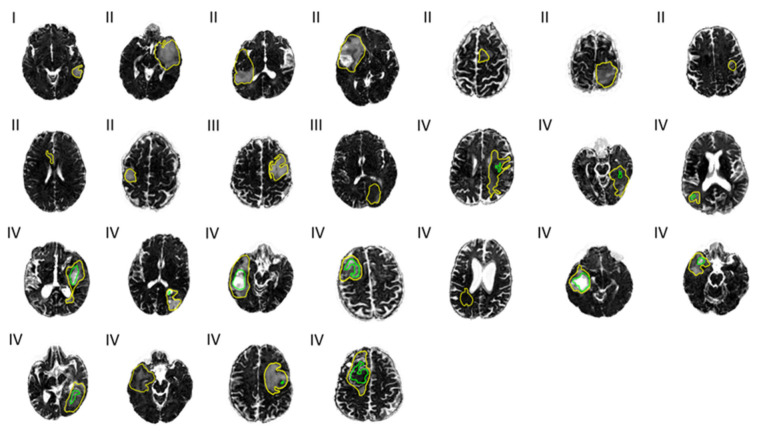
Free water (*FW*) maps across 25 patients. Tumor grades I, II, III, and IV are noted to the top left at each map. Outlines of the total tumor regions (yellow) and necrotic tumor regions (green) are overlaid.

FW maps with cross-sections of total and necrotic tumor regions overlaid can be seen in Figure 3. The variance in mean *FW*, as mentioned above, may be visually suggested by the varying gray scale levels representing high and low FW content within the necrotic areas (green outline).

The *FA* distribution in the necrotic tumor region is least affected by FW corrections, as there is no significant change in *FA* values, as measured by the mean, median, and quantiles (Table 2). In non-necrotic regions, these summary variables increase on average by 20.4%, and kurtosis decreases by 56.6%. Entropy increases by 6.7% in necrotic areas and on average by 7.5% in non-necrotic regions. Skewness decreases by 49.9% in necrotic areas and on average by 62.2% in non-necrotic regions.

**Figure 4:**
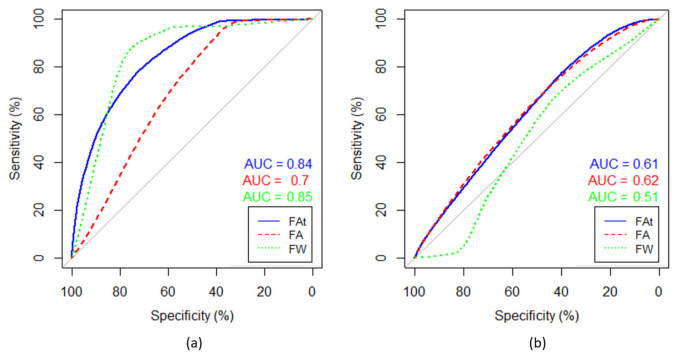
Receiver operator characteristics (ROCs) based on voxel-wise labelling. (**a**) ROC curves separating necrotic and enhancing tumor regions, and (**b**) ROC curves separating enhancing and non-enhancing tumor regions. Enhancing tumor region is defined as the true positive and the adjacent necrotic tumor region as the true negative. The patient is the same as the one shown in all single patient examples in this paper. The data were fitted to a logistic regression model and the ROC was computed for free-water (FW)-corrected fractional anisotropy (*FA*) (blue), non-corrected *FA* (red), and *FW* (green). Area under curve (AUC) is rendered in corresponding colors.

ROC curves were calculated based on voxel-wise labelling of necrotic and enhancing tumor regions and a logistic regression model and show that necrotic tumor regions can be better differentiated using FW-corrected data than with regular *FA* maps. Results are shown from a single patient in Figure 4. In this patient, the average area under the curve (AUC) is greater than average. Across all patients, the AUC was 0.77 based on *FAt*, 0.73 with *FA*, and 0.77 with *FW*. Enhancing tumor regions were defined as the true positive and the adjacent necrotic tumor regions as the true negative. When enhancing tumor regions are separated from the adjacent non-enhancing tumor regions and non-enhancing tumor regions are labelled as the true positive, the AUCs are 0.66, 0.67, and 0.62 in *FAt*, *FA*, and *FW*, respectively.

### 3.2. Impact of FW Correction on Parameter Distributions

**Figure 5:**
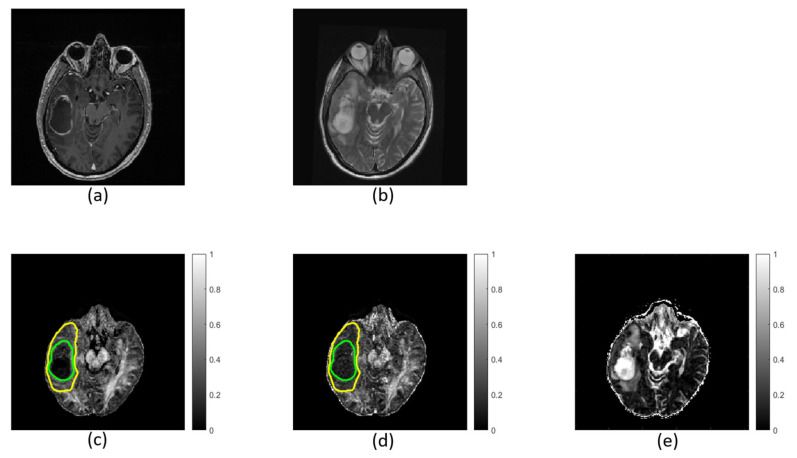
Images and maps from a grade IV patient: (**a**) T_1_w image; (**b**) T_2_w image; (**c**) free-water (FW)-corrected fractional anisotropy (*FAt*) map; (**d**) non-corrected fractional anisotropy (*FA*) map with contours defining non-enhancing tumor; and (**e**) FW map. Within the non-enhancing region, more heterogeneity is revealed in the *FAt* map compared to the *FA* map. The FW map quantitatively highlights FW content.

FW elimination increases *FA* entropy (*p* ≤ 0.003) and decreases *FA* skewness (*p* ≤ 0.004) in the non-enhancing regions in both low- and high-grade tumors (Table 3). Kurtosis does not decrease significantly in low-grade tumors (*p* = 0.90) but does decrease significantly in high-grade tumors (*p* ≤ 0.001). The *FA* distribution shifts toward higher *FA* values after FW correction. Refer to the Supplementary Material for a complete table of summary variables (Supplementary Material, Table S1). Maps calculated with and without FW correction are shown for a single patient in Figure 5.

No significant correlation was found between tumors *FW, FAt,* and *FA* (Supplementary Material, Table S2) nor to any parameter distribution summary variables apart from the mean (variance, 25th and 75th quantile, median, entropy, kurtosis, and skewness).

## 4. Discussion

Improved oncological imaging is dependent on an accurate description of tissue microenvironments, and descriptions of tumor heterogeneity may be improved by correcting for FW. The aim is to better characterize glioma by using FW modeling. Multi-shell DTI fitting to estimate and remove the FW contribution was performed using the method developed by Pasternak et al. [18]. The results indicate that by removing fluid bias from conventional *FA* maps, FW estimation and elimination allow for a quantitative non-invasive characterization of glioma, and FW correction improved automatic labelling of necrotic tumor voxels. Results show that FW correction significantly increases entropy across grades and significantly reduces kurtosis in high-grade tumors. Increased entropy may imply greater detected heterogeneity.

### 4.1. Characterizing Tumor Sub-Regions

*FW* maps show large variation of FW content within necrotic tumor regions across patients, suggesting that FW estimations can be an easily applicable method for quantitatively characterizing necrotic regions, as there is a reduction of cell density with necrosis [26,27]. Typically, lower grade patients do not have significant necrotic regions, but they may develop with progressing disease. Necrosis can be detected based on expert evaluations of MRI (post-contrast T_1_w images in particular), but quantitative thresholds of FW content to characterize necrotic regions are not routinely applied in the clinic. Some studies have suggested that a more accurate description of the necrotic region helps with predicting survival and tumor aggressiveness in glioblastoma patients [40,41], and deep learning-based segmentation [42] and tumor classification [43] are being explored. Adding the estimation of *FW* to such analyses is a possible, clinically useful application of FW estimation. Conventional *MD* that is not corrected for FW could not significantly differentiate between tumor regions. Calculating *FW* content may therefore add to a more precise description of the tumor.

The *FA* distribution within grade IV tumors changed significantly in all parts of the tumor region, except in the necrotic tumor region. Significant differences between *FA* and *FAt* distributions were not present in FW correction of the necrotic tumor region as expected. The necrotic region contains large amounts of water, and due to the short *TR* used in this study, a concern could be incomplete signal spoiling in these regions. However, the diffusion signal versus b-values had the expected exponential decay (data not shown), confirming that the acquisition protocol did not bias the results. The DWI signal decay in both ventricles and the two most cystic necrotic volumes was found to be normal, and *FW* was estimated to one in both cases. It is possible that FW correction methods, such as the one applied in this study, can be of value in differentiating true disease progression from pseudo-progression [44]. Tumor cellularity, as derived with RSI, has been presented as a possible diagnostic tool in identifying pseudo-progression in glioblastoma [45,46]. The patients in this study are treatment-naïve; consequently, no pseudo-progression can be measured. Pseudo-progression is often accompanied by dead tissue and water, or it is due to immune cell infiltration [47]. Since the *FA* distribution changed significantly in enhancing and non-enhancing tumor regions and not in necrotic regions of these treatment-naïve patients, it is possible that pseudo-progression might be detected with FW correction in the enhancing and non-enhancing regions, if it could in the future be demonstrated that the *FA* distribution is also different in regions containing less structure due to dead tissue, water, or immune cell infiltration.

ROCs separating necrotic from enhancing tumor volumes had larger AUCs with applied FW correction than without. This implies that FW-corrected data may reduce partial volume effects, present on regular DWI, even if the tissues in enhancing volumes and non-enhancing volumes are not very different. We found no statistically significant differences between enhancing and non-enhancing tumor volumes based on *FA* or *FW*. It is important to note that ROC comparisons were performed with reference to regular structural T_1_w and T_2_w MR images and not histologically confirmed pathology. The logarithmic fits performed as part of the ROC curve calculations are relatively poor. This is because of the parameters *FW, FAt,* and *FA* to some extent overlapping in, for example, necrotic and enhancing tumor regions. However, the point is not to fit the data to a logarithmic model, but merely to calculate a parameter cut-off point that provides a basis for a mathematical evaluation of ROC predictions. Based on “Restricted Spectrum Imaging” (RSI)-based tissue modelling, it has previously been demonstrated that accounting for FW components has been shown to help with tumor segmentation [48]. However, the study by White et al. [48] focused on the delineation between tumor and normal-appearing white matter based on various intensity ratios of ADC within tumor regions and normal-appearing white matter and not on calculations of *FW* or *FAt*.

### 4.2. Impact of FW Correction on Parameter Distributions

The impact of free water elimination was found to significantly influence ROI summary variables, regardless of tumor grade when compared to regular diffusion tensor metrics. Entropy of *FA* increases in the tumor volume, which means that tissue heterogeneity may reflect the underlying heterogeneity better, helping in the assessment of the tumor microenvironment. Higher mean, median, and 25th and 75th quantiles in the FW-corrected maps show that the mass of the distribution is shifted towards higher *FA* values. There is an increase in median, quantiles, and mean *FA* after removal of FW contamination in the measured signal. These changes are expected when the isotropic signal is removed by the employed FW-correction method. Kurtosis and skewness decrease after FW correction, and the *FAt* distribution comes closer to a normal distribution than the uncorrected *FA* distribution. This can be an effect of the increasing summary variables or an indication that varying levels of FW are corrected for across the volume. *FAt* maps have higher entropy than *FA* maps, which may be indicative that FW correction reveals tissue heterogeneity. This heterogeneity may be due to, for example, fiber tracts or infiltrative tumor in the peritumoral tissue.

In our study, we were not able to identify *FW, FAt,* or *FA* distribution summary variables (mean, variance, 25th and 75th quantiles, median, entropy, kurtosis, or skewness) that correlated with tumor grades. This adds to inconsistent findings in previous studies, where some studies reported increased *MD* in high-grade glioma [8], while others reported decreased *MD* [9,49] or no difference in *MD* [11]. Since *MD* was used for initialization of the FW correction in our study, we do not report on *MD*. Similar to *MD*, *FA* has also been found to be increased [49], decreased [8], and not significantly different [9,11] across tumor grades, as well as to be increased in areas with recurrent tumors [44]. Kurtosis decreases in non-enhancing areas in high-grade tumors and not in low-grade tumors. This may be due to a larger number of low-grade tumors not having significant edema.

### 4.3. Limitations

FW correction provides more detail of the structures that are otherwise hidden beneath the edema, though apart from better characterization of the tumor regions, further studies are required to test the benefits of the FW correction in gliomas. Although tumor sub-regions were drawn on structural T_1_w and T_2_w MR images by an expert radiologist, the standard against which the FW-correction-based differentiation between tumor sub-regions were assessed should ideally be the pathology confirmed by histology. The method employed in our study makes use of multiple b-shell acquisitions. Nevertheless, it is still easily applicable in the clinic routine considering the limited increase in acquisition time. This study is a proof of concept, using previously acquired prospective data; hence, the sample size is limited. Another limitation is the quality of the diffusion-weighted imaging performed. Due to the relatively small number of signal acquisition from only eight diffusion directions, the *FW* and *FA* maps, as well as the DWI used for fiber tract reconstruction, are noisier than they could have been with more directions and signal averaging. However, the data represented in this study only took 4 min to acquire; therefore, it represents a clinically feasible scenario.

## 5. Conclusions

In conclusion, *FW* estimation is a way to quantitatively and non-invasively characterize necrotic tumor regions in particular. Monitoring potential changes within tumor regions due to developments of the disease may also be possible, though the hypothesis produced by these results must be further evaluated by histology of FW-mapped regions. Results based on *FA* maps of the tumor region are significantly impacted by FW correction, suggesting increased heterogeneity. The applied method is easily applicable in the clinic and reveals potentially important physiological information.

## Figures and Tables

**Table 1:** Number of patients, mean total tumor volume, mean age, and sex across tumor grades. Note that edema was included in total volume, and the single grade I patient did not have much edema.

	Grade I	Grade II	Grade III	Grade IV
Number of patients	1 (1 F *)	8 (7 F, 1 M **)	2 (1 F, 1 M)	15 (6 F, 9 M)
Mean age	22 years	35 ± 11 years	46 ± 11 years	65 ± 10 years
Mean total tumor volume	6.0 cc ***	57.6 ± 76.5 cc	48.0 ± 12.5 cc	46.7 ± 27.2 cc

* F = female, ** M = male, *** cc = cubic centimeters.

**Table 2:** Significances of the effects of free water correction on fractional anisotropy (*FA*) summary variables across tumor regions (TR) in grade IV patients. *p*-values are calculated with a paired *t*-test and a significance threshold of *p* < 0.05. The *p*-values are adjusted for multiple comparisons using the Bonferroni method.

*p*-Values	Enhancing TR	Necrotic TR	Non-Enhancing TR	Total TR
Mean	0.013	1.0	<0.001	<0.001
Variance	0.11	1.0	1.0	1.0
25th Quantile	<0.001	0.27	<0.001	0.001
75th Quantile	0.005	0.068	<0.001	<0.001
Median	<0.001	0.22	<0.001	<0.001
Entropy	0.010	0.018	<0.001	<0.001
Kurtosis	<0.001	0.50	0.003	<0.001
Skewness	<0.001	0.018	<0.001	<0.001

**Table 3:** Comparison of fractional anisotropy summary variables with and without free water corrections in the non-enhancing tumor region. Reported *p*-values were computed according to the paired *t*-test and were adjusted with the Bonferroni method (significance threshold < 0.05).

Summary Variables from the Non-Enhancing Tumor Volume		Grade I and II (n = 9)		Grade III and IV (n = 16)	
		Mean and std. dev	*p*-Value	Mean and std. dev	*p*-Value
Entropy	FAt *	7.21 ± 0.31	0.003	7.15 ± 0.15	<0.001
	FA **	6.73 ± 0.47		6.67 ± 0.35	
Kurtosis	*FAt*	3.63 ± 1.44	0.90	3.57 ± 0.89	<0.001
	*FA*	7.86 ± 5.03		7.22 ± 3.76	
Skewness	*FAt*	0.68 ± 0.51	0.004	0.59 ± 0.32	<0.001
	*FA*	1.58 ± 0.73		1.43 ± 0.71	

* *FAt* = free-water-corrected fractional anisotropy, ** *FA* = non-corrected fractional anisotropy.

## Data Availability

All data are available upon reasonable request and according to the funding sources’ policies from the corresponding author. Requests for materials should be addressed to F. R.

## Supplementary Materials

The following are available online at https://www.mdpi.com/article/10.3390/diagnostics11122385/s1, Figure S1: Conventional MD from non-corrected data. The first four box and whisker plots from the left show the mean MD in enhancing tumor regions, necrotic tumor regions, non-enhancing tumor regions, and total tumor regions from grade IV patients. The fifth box and whisker plot shows the mean parameter values in total tumor regions from all patients. No statistical difference was found between tumor regions. Statistics were performed with ANOVA tests and Tukey’s post hoc test at a significant threshold of *p* < 0.05. Table S1: Comparison of fractional anisotropy summary variables with and without free water corrections in the non-enhancing tumor volume. Reported *p*-values were computed according to the paired t-test and were adjusted with the Bonferroni method (significance threshold < 0.05), Table S2: Comparison of fractional anisotropy summary variables between low- and high-grade non-enhancing tumor volumes with and without free water corrections. Reported *p*-values were computed according to the unpaired *t*-test and were adjusted with the Bonferroni method (significance threshold < 0.05).
