# Supplementary material for: Effects of Multi-Shell Free Water Correction on Glioma Characterization
Source: Diagnostics (Basel). 2021 Dec 17;11(12):2385. doi: 10.3390/diagnostics11122385 (PMC8700586; doi:10.3390/diagnostics11122385)
Supplement: Supplementary file 1 [file diagnostics-11-02385-s001.zip › diagnostics-1493557-supplementary.pdf]

Supplementary Material

Characterizing tumor sub-regions

Although there may be tendencies toward higher *MD* in the necrotic region compared to non-necrotic regions, conventional *MD* that is not corrected for FW does not significantly differentiate between tumor regions (Figure S1).

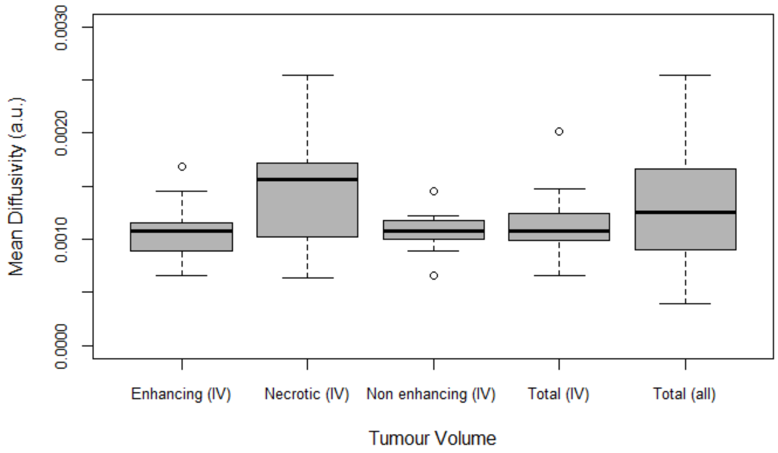

Figure S1: Conventional *MD* from non-corrected data. The first four box and whisker plots from the left show the mean *MD* in enhancing tumor regions, necrotic tumor regions, non-enhancing tumor regions, and total tumor regions from grade IV patients. The fifth box and whisker plot shows the mean parameter values in total tumor regions from all patients. No statistical difference was found between tumor regions. Statistics were performed with ANOVA tests and Tukey's post hoc test at a significant threshold of $p < 0.05$.

Comparison of *FA* to *FAt* measures

The effect of FW-correction on low-grade (grade I and II) tumors and high-grade (grade III and IV) tumors were investigated using paired t-tests. *FAt* was higher than *FA* (Table S1), as reflected by significantly increased means, and quantiles ($p \leq 1.3 \cdot 10^{-5}$). Entropies are higher in *FAt* distributions ($p \leq 3.0 \cdot 10^{-3}$). The *FAt* summary variables also had statistically significantly reduced skewness ($p \leq 4.0 \cdot 10^{-3}$). In high grade tumors, kurtosis was significantly reduced ($p = 3.1 \cdot 10^{-4}$). The effects of FW correction were similar in both the low- and high-grade groups, although reduction of kurtosis was not found to be significant in the low-grade group.

Summary variables from the non-enhancing tumor volume		Grade I and II (n = 9)		Grade III and IV (n = 16)	
		**Mean and std. dev**	**p-value**	**Mean and std. dev**	**p-value**
Mean	*FAt*	0.29 ± 0.07	**<0.001**	0.32 ± 0.06	**<0.001**
	FA	0.22 ± 0.06		0.25 ± 0.05	
Variance	*FAt*	0.01 ± 0.01	1.0	0.014 ± 0.004	0.59
	FA	0.02 ± 0.01		0.015 ± 0.005	
25th quantile	*FAt*	0.20 ± 0.04	**<0.001**	0.23 ± 0.05	**<0.001**
	FA	0.13 ± 0.03		0.17 ± 0.04	
75th quantile	*FAt*	0.37 ± 0.10	**<0.001**	0.39 ± 0.07	**<0.001**
	FA	0.27 ± 0.09		0.31 ± 0.06	
Median	*FAt*	0.28 ± 0.07	**<0.001**	0.30 ± 0.06	**<0.001**
	FA	0.19 ± 0.05		0.23 ± 0.05	
Entropy	*FAt*	7.21 ± 0.31	**0.003**	7.15 ± 0.15	**<0.001**
	FA	6.73 ± 0.47		6.67 ± 0.35	
Kurtosis	*FAt*	3.63 ± 1.44	0.9	3.57 ± 0.89	**<0.001**
	FA	7.86 ± 5.03		7.22 ± 3.76	
Skewness	*FAt*	0.68 ± 0.51	**0.004**	0.59 ± 0.32	**<0.001**
	FA	1.58 ± 0.73		1.43 ± 0.71	

FAt = Free water Corrected Fractional Anisotropy, *FA* = Non-corrected Fractional Anisotropy

Table S1: Comparison of fractional anisotropy summary variables with and without free water corrections in the non-enhancing tumor volume. Reported p-values are computed according to the paired t-test and are adjusted with the Bonferroni method (significance threshold < 0.05).

Free water correction and tumor grading

Unpaired t-tests to compare fractional anisotropy summary variables between low- and high grade non-enhancing tumor regions with and without free water corrections. No significant differences were found between low-grade (grade I and II) and high grade (grade III and IV) in either *FAt* or *FA* distribution summary variables (Table S2).

Summary variables from non-enhancing tumor volume		Grade I and II (n = 9)	Grade III and IV (n = 16)	p-value
		Mean and std. dev	Mean and std. dev	
Mean	*FW*	0.32 ± 0.08	0.30 ± 0.09	1.0
	FAt	0.29 ± 0.07	0.32 ± 0.06	0.37
	FA	0.22 ± 0.06	0.25 ± 0.05	0.16
Variance	*FW*	0.02 ± 0.01	0.02 ± 0.01	1.0
	FAt	0.01 ± 0.01	0.014 ± 0.004	0.39
	FA	0.02 ± 0.01	0.015 ± 0.005	1.0
25th quantile	*FW*	0.24 ± 0.09	0.19 ± 0.06	1.0
	FAt	0.20 ± 0.04	0.23 ± 0.05	0.64
	FA	0.13 ± 0.03	0.17 ± 0.04	0.16
75th quantile	*FW*	0.41 ± 0.09	0.38 ± 0.13	1.0
	FAt	0.37 ± 0.10	0.39 ± 0.07	1.0
	FA	0.27 ± 0.09	0.31 ± 0.06	1.0
Median	*FW*	0.32 ± 0.09	0.29 ± 0.10	1.0
	FAt	0.28 ± 0.07	0.30 ± 0.06	1.0
	FA	0.19 ± 0.05	0.23 ± 0.05	0.64
Entropy	*FW*	7.11 ± 0.31	7.04 ± 0.46	1.0
	FAt	7.21 ± 0.31	7.15 ± 0.15	1.0
	FA	6.73 ± 0.47	6.67 ± 0.35	1.0
Kurtosis	*FW*	4.01 ± 1.44	5.44 ± 4.31	1.0
	FAt	3.63 ± 1.44	3.57 ± 0.89	1.0
	FA	7.86 ± 5.03	7.22 ± 3.76	1.0
Skewness	*FW*	0.15 ± 0.76	0.89 ± 0.89	0.32
	FAt	0.68 ± 0.51	0.59 ± 0.32	1.0
	FA	1.58 ± 0.73	1.43 ± 0.71	1.0

FW = Fractional Volume of the Free Water compartment, *FAt* = Free water Corrected Fractional Anisotropy, *FA* = Non-corrected Fractional Anisotropy.

Table S2: Comparison of fractional anisotropy summary variables between low- and high grade non-enhancing tumor regions with and without free water corrections. Reported p-values are computed according to the unpaired t-test and are adjusted with the Bonferroni method (significance threshold < 0.05).
